# Fermentative Yeast Diversity at the Northern Range Limit of Their Oak Tree Hosts

**DOI:** 10.1111/1758-2229.70110

**Published:** 2025-05-23

**Authors:** Javier Pinto, Chloé Haberkorn, Markus Franzén, Ayco J. M. Tack, Rike Stelkens

**Affiliations:** ^1^ Department of Zoology Stockholm University Stockholm Sweden; ^2^ Department of Biology and Environmental Science, Center for Ecology and Evolution in Microbial Model Systems Linnaeus University Kalmar Sweden; ^3^ Department of Ecology, Environment and Plant Sciences Stockholm University Stockholm Sweden

**Keywords:** biodiversity, climate data, distribution patterns, DNA metabarcoding, fermentative yeast, northern range limit, oak

## Abstract

Fermentative yeasts play important roles in both ecological and industrial processes, but their distribution and abundance in natural environments are not well understood. We investigated the diversity of yeasts at the northern range limit of their oak tree hosts (*Quercus* spp.) in Sweden, and identified climatic and ecological conditions governing their distribution. Yeasts were isolated from bark samples from 28 forests and identified to the species level using DNA metabarcoding. Most communities were dominated by species in the Saccharomycetaceae family, especially by species of *Saccharomyces, Kluyveromyces* and *Pichia*. Each genus showed a distinct latitudinal and longitudinal distribution, and both temperature and precipitation metrics predicted significant variation in their abundance. Consistent with this, laboratory assays revealed significant effects of temperature on the growth of strains collected from different longitudes and latitudes. We found that older trees harbour more diverse and more balanced fermentative yeast communities with more evenly distributed species abundances. Communities across trees were more similar when sharing a common dominant species. This work provides a baseline for future studies on the impact of climate change on the fermentative yeast biodiversity of temperate forests in northern latitudes and contributes to a growing collection of wild isolates for potential biotechnological applications.

## Graphical abstract

Fermentative yeast species dominance correlated to environmental gradients.

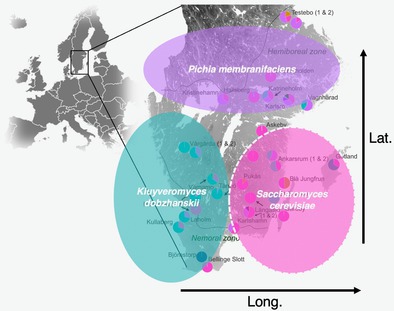

## 1 Introduction

Fermentative yeasts are abundant in temperate forests where they play a crucial role in ecological processes (Mozzachiodi et al. 2022). They recycle organic matter, mediate nitrogen and carbon cycles and serve as a food source for insects (Botha 2011). They also impact microbial community composition, for example, by inhibiting the growth of competing microorganisms by producing ethanol (Viljoen 2006). Fermentative yeasts are also known for their high stress tolerance and their efficient use of monosaccharides and nitrogen (Treseder and Lennon 2015; Romero‐Olivares et al. 2021), making them well‐suited for industrial applications, particularly, for the production of alcoholic beverages and biofuels. Recent studies suggest that the metabolic diversity of natural yeasts, their variation in niche breadth and stress tolerance, is largely shaped by genetic factors (Opulente et al. 2024). A recent global distribution analysis showed that species richness is highest in mixed, montane forests in temperate climates and is best predicted by microhabitat, vegetation type and topography (David et al. 2024). Species ranges are strongly influenced by overlaps with other yeast species, indicating that niche partitioning plays an important role in their biogeography. The distribution of budding yeasts in the genus *Saccharomyces* is known to be associated with environmental temperature (David et al. 2024; Mozzachiodi et al. 2022; Sweeney et al. 2004).

Oak trees (*Quercus* spp.) have been described as a frequent habitat for fermentative yeasts in different parts of the world (Mozzachiodi et al. 2022; Robinson et al. 2016; Sampaio and Gonçalves 2008; Sniegowski et al. 2002). Oak trees, especially their bark and sap, provide natural sugars (Ferreira et al. 2018) and can accumulate decaying plant matter and moisture, which creates suitable growth environments for yeasts. Once the polysaccharides in the bark are broken down into smaller sugars, for example, by filamentous fungi (Battaglia et al. 2011; de Vries and Visser 2001), they are available as a carbon source for fermentative yeasts. Forests in the north of Europe represent the northern range limit of oak, which is marked by long, cold winters and warm (but not hot) summers, a shorter growing season and more coniferous vegetation. Historically, oak forests were widespread in southern Scandinavia. Over the past centuries, a combination of biogeographical shifts and human activities have significantly reduced the volume of oak forests (Löf et al. 2016). Tree health has also substantially deteriorated, especially in *Quercus robur* , which has suffered from crown defoliation and a general decline in recent decades (Drobyshev et al. 2007). Today, the remaining oak forests are concentrated along the coastlines in southern Sweden in the temperate and hemiboreal climate zones, delimited in the north by the subarctic climate of the boreal zone (Drobyshev et al. 2008). Although remaining oak forests in Sweden are likely important reservoirs of microbial diversity, the current lack of microbial data has precluded a systematic investigation of this ecosystem. Whether fermentative yeasts are part of the oak microbiome also in the northernmost range of the *Quercus* distribution is so far unknown.

Our overarching aim was to investigate the diversity of fermentative yeasts found in the northern range limit of oak and identify climatic and environmental drivers of their distribution. For this, we isolated yeasts from oak bark samples collected across southern Sweden, specifically from the nemoral and hemiboreal climate zones (Jonsson et al. 2016). Our sampling area covered 583 km from south to north, along latitudes between 55°52′33″ N and 60°76′78″ N (from southern Sweden to as far north as the Swedish oak tree line allowed) and 379 km from west to east, along longitudes between 12°48′38″ E and 18°63′15″ E (from the North sea to the Baltic sea). We used enrichment protocols designed for fermentative yeasts in the genera *Saccharomyces*, *Komagataella*, *Lachancea* and *Candida* (Cubillos et al. 2019; Sampaio and Gonçalves 2008; Sniegowski et al. 2002; Villarreal et al. 2022). For biodiversity and abundance assessment, we used metabarcode sequencing of the Internal Transcribed Spacer 1 (ITS1) region, widely used in fungal community profiling, particularly, within Ascomycota (Blaalid et al. 2013; Tedersoo et al. 2022), which includes most of the targeted fermentative yeasts. Furthermore, it allows identification of yeasts down to the species level (Alsammar et al. 2019; Větrovský et al. 2019).

Specifically, we asked (1) if communities of fermentative yeasts differ in their composition and diversity, (2) if there are spatial patterns in fermentative yeast community structure along latitudinal and longitudinal gradients, reflecting variation in temperature and precipitation, (3) if oak tree host characteristics (e.g., tree age) predict yeast diversity and (4) if the growth of the yeast strains we isolated is affected by temperature, depending on the location they were isolated from, using laboratory assays.

## 2 Materials and Methods

### 2.1 Yeast Sampling From Oak Tree Bark

**FIGURE 1:**
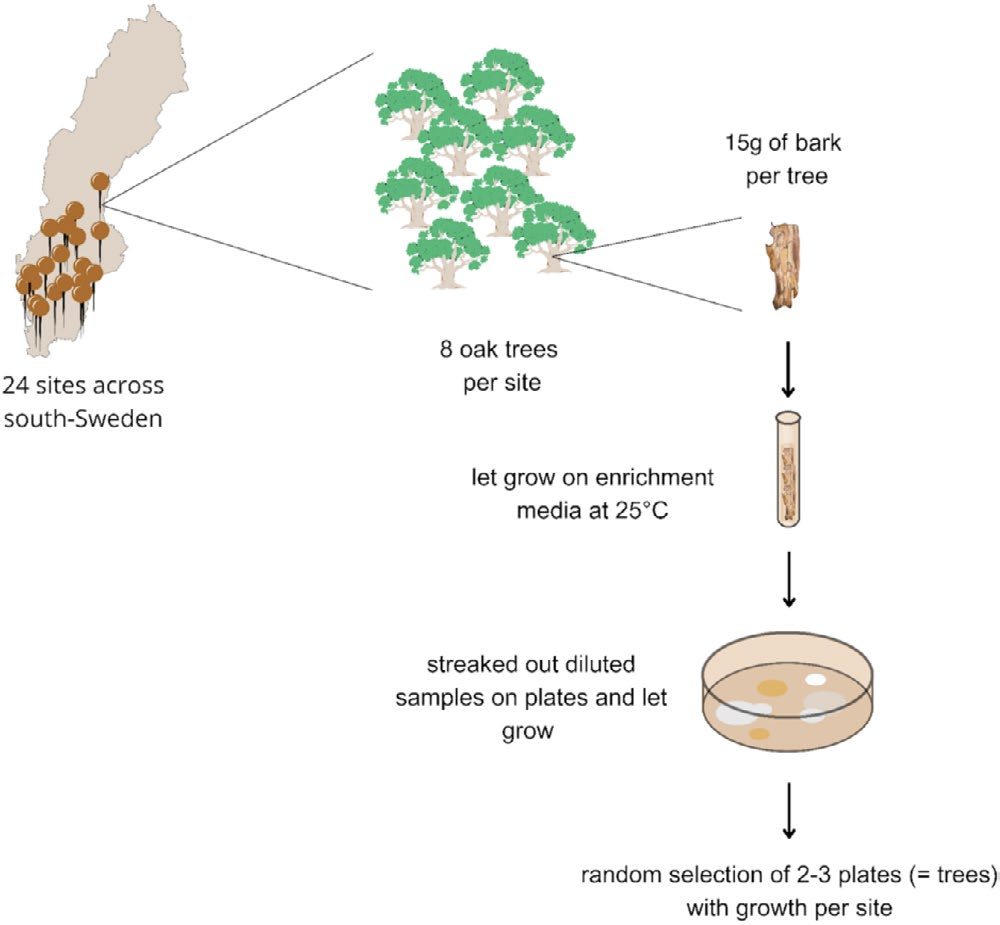
Schematic of the sampling, enrichment and isolation of yeast from oak trees.

Eight oak trees were sampled in each of 28 stands, at 23 different locations across the south of Sweden, in both nemoral and hemiboreal vegetation zones (Figure 1). Trees included 67 *Q. robur*, nine *Quercus petraea* and oak trees (*Q. sp*) with unidentified species status. The sampling period was during the mid and late summer, from July to September 2023. Approximately 15 g of bark (ca. 4 × 1 × 0.5 cm), including both the outer and inner bark layers, was collected from each tree using an ethanol‐sterilised knife. Where single pieces of this size were not obtainable, multiple smaller bark pieces were collected. Samples were collected using nitrile gloves and stored individually in sterile plastic bags. All sampling tools were sterilised with 90% ethanol before and after sampling. Samples were transported in a cooled container and stored refrigerated until processing.

Bark samples from each tree were grown in 25 mL of liquid enrichment medium (a modified version of the medium described by Sampaio and Gonçalves (2008)), consisting of YNB (yeast nitrogen base) supplemented with 1% (wt/vol) raffinose and 4% (vol/vol) ethanol. While in the original protocol 8% ethanol was used, we reduced it to 4% ethanol to increase our chances of uncovering more biodiversity, including groups of yeasts that are not able to tolerate high alcohol concentrations. We inoculated this enrichment medium with bark samples for 15 days, in 50 mL Falcon tubes at room temperature. From each tube, 100 μL of liquid was then sampled and diluted 4× in sterilised water. Fifty microliters of diluted sample were then plated onto solid YMA medium (1% glucose, 0.5% peptone, 0.3% yeast extract, 0.3% malt extract, 2% agar) with 4% ethanol using glass beads to spread cells evenly onto solid medium and grown for 5 days at 30°C. To confirm the presence of yeasts, a PCR of the whole ITS region was performed for three yeast colonies growing on an agar plate per stand. Two to three individual trees per stand with growth of putative yeasts were then randomly selected for DNA extraction (total number of trees, *n* = 81).

### 2.2 DNA Extraction

From each enrichment plate (one per tree) we haphazardly selected ~20 colonies, representative of all colony types visible to the bare eye, by scraping the colonies with a sterile pipette tip and adding them to 250 μL of 1× PBS. After vortexing, samples were spun down at 3000 rpm for 3 min and the supernatant was discarded. Fifty microliters of zymolyase solution (1 mg/mL zymolyase, 1 M sorbitol, pH 7) were added to the pellet and mixed by pipetting. Cells were then incubated at 37°C for 2 h before adding 1 mL of sterilised water. Cells were then spun down at 13,000 rpm for 30 s to discard the supernatant and resuspended in 250 μL PBS. After vortexing, 20 μL of proteinase K solution and 290 μL of AL buffer were added to each sample and mixed thoroughly by pipetting. Samples were then incubated at 60°C for 20 min and cellular debris was removed by centrifugation at 3000 rpm for 3 min. A KingFisher 96 deep‐well plate was prepared following the OMEGA Bio‐Tek protocol to perform the DNA extraction. PCR1 was performed to enrich the samples for putatively present yeast‐DNA, by amplifying ITS1 (using ITS1 and ITS2 primers, sequences in Table S1) (White et al. 1990; Alsammar et al. 2019). Amplification was carried out using the Takara Taq polymerase kit. PCR1 amplicon concentrations were assessed using Qubit dsDNA HS Assay Kits. All concentrations were normalised to 2 ng/μL by diluting samples in MilliQ water, up to a volume of 50 μL per sample.

### 2.3 Sequencing

PCR1 amplicons with normalised concentrations were cleaned up using MagSI‐DNA NGS PREP Plus magnetic beads at the National Genomics Infrastructure (NGI, Solna, Sweden), followed by an indexing PCR2 with Adaptamera indices and a second cleanup using magnetic beads. Quality control was performed on the final library before sequencing on the NovaSeq 6000 platform (Illumina NextSeq, 2 × 300 bp paired‐end reads). Raw reads were deposited in NCBI's sequence read archive (SRA) database under BioProject PRJNA1114957.

### 2.4 Read Processing

Reads were processed to identify yeast species using the nf‐core/ampliseq pipeline (Straub et al. 2020) with default parameters and option ‘illumina_pe_its’. Amplicon sequence variants (ASVs), that is, unique DNA sequences obtained from amplicon sequencing, were inferred and annotated with DADA2 against the UNITE general FASTA release for Fungi v9.0 (Abarenkov et al. 2024). Species names were verified using the website MycoBank (Robert et al. 2013), accessed in April 2025, to cross‐check names extracted from Fungi v9.0 (updated names were: *Issatchenkia orientalis* > *Pichia kudriavzevii*; *Kazachstania servazzii* > *Monosporozyma servazzii*; *Candida castelli* > *Oligophagozyma castellii* and *Debaryomyces delbrueckii* > *Torulaspora delbrueckii*). We then filtered out ASVs that were below a confidence threshold of 97% similarity to UNITE reference sequences (Nilsson et al. 2019), reducing the total number of ASVs from 353 to 149 ASVs. Species‐level identifications were inferred from the 149 high‐confidence ASVs. All the scripts used for data processing, analyses and figures are openly available on GitHub: https://github.com/chaberko‐lbbe/yeast‐oaktree.

### 2.5 Environmental Predictors

Longitudes and latitudes were extracted from GPS coordinates for each sampling location. Using these coordinates, temperature and precipitation data were extracted from WorldClim version 2.1 (Fick and Hijmans 2017) with QGIS software (Geographic Information System).

We used monthly averages to calculate annual mean temperature, temperature during the coldest month (ranging from −8°C to −2°C) and temperature during the warmest month (ranging from 22°C to 25°C) at each location. Because mean temperature was strongly correlated with temperature during the coldest month, we only kept temperature during the coldest month and temperature during the warmest month. For rainfall, we used the monthly averages to determine rainfall during the driest month (ranging from 13.3 to 32.5 mm) and rainfall during the wettest month (ranging from 67.9 to 136.7 mm) at each location.

### 2.6 Individual Host Tree Metrics

Tree height was measured using a Suunto PM‐5/1520 clinometer. Diameter at 1.3 m breast height (DBH) was assessed using Haglöf Mantax Blue Klave callipers. Bark depth was measured as the mean depth of bark crevices in the four cardinal directions (North, South, East and West) using a metal ruler, following the methodology of Johansson et al. (2009). Tree age was determined by counting the total number of annual growth rings in each core sample obtained at breast height, following standard dendrochronological procedures and cross‐validation (Holmes 1983). This provided a minimum age estimate, as core sampling at 1.3 m height does not account for the time required for trees to reach breast height. Annual radial growth rates were quantified through tree‐ring widths (TRWs) measurements using a digital LINTAB positioning table connected to an Olympus stereomicroscope. These measurements were recorded to the nearest 0.01 mm using TSAPWin Scientific software (Rinn 2003). Each annual ring width represents the radial stem increment for that particular year, providing a high‐resolution time series of growth patterns. Growth rates were calculated as the annual increment in ring width, with the mean annual growth rate determined by averaging these measurements across the entire core sample. To account for age‐related growth trends and ensure data quality, all TRW series underwent standardisation and cross‐dating procedures using COFECHA software (Johnson and Abrams 2009).

### 2.7 Growth‐at‐Temperature Assays

To test whether temperature has an effect on the growth of the strains we isolated from the wild, we assessed the species affiliation of three colonies per site by Sanger sequencing. Since the strains were collected blindly from the remaining colonies on the plates after amplicon sequencing, some did not match the dominant species identified for each location by amplicon sequencing. Thus, for the growth assays, we only retained strains identified by Sanger sequencing as *Saccharomyces paradoxus*, *Kluyveromyces dobzhanskii* and *Pichia membranifaciens*, from a total of 18 locations. Sanger sequencing confirmed the presence of *P. membranifaciens* in only one sample (Halmstad 1), which precluded us from testing for effects of latitude and longitude on growth‐at‐temperature for this species.

We measured the maximum biomass of each strain as a proxy for growth at three different temperatures: 5°C (below 4°C growth is usually no longer observed in yeasts; Salvadó et al. 2011), 16°C, which is close to the mean temperature during Swedish summer; and 35°C, which is close to the highest temperature ever recorded in Sweden (SMHI 2023). Growth assays were performed on a BioTek Epoch 2 microplate spectrophotometer (Agilent, USA) using maximum biomass, measured as optical density (OD_600nm_). We standardised all inoculates to an initial OD_600nm_ of 0.1 (approximately 10^6^ cells) in 200 μL of YPD medium (20 g/L peptone, 10 g/L yeast extract, 2% glucose). Before final growth measurements were taken, each strain was grown for 24 h (for measurements at 16°C and 35°C) and for 120 h (for measurements at 5°C) in 96‐well plates. We used 10 technical OD replicates per strain. Biomass was blank‐corrected using negative controls (six wells with medium but no yeast).

### 2.8 Statistical Analysis

Abundance and taxonomy tables based on the read counts associated with ASVs were used to compute alpha diversity indices: ASV richness (the number of different ASVs), Shannon (accounting for both ASV richness and the proportional abundance of each ASV within a sample; Shannon and Weaver 1963) and Evenness indices (how evenly distributed ASV abundance is among species within a community; Wilsey and Potvin 2000) with the package R/vegan v2.5‐7 (Oksanen et al. 2020). A non‐metric multidimensional scaling (NMDS) ordination was computed based on ASV richness using the vegan function metaMDS (distance ‘bray’, two dimensions, try max 1000). To evaluate whether pairs of species co‐occur more or less frequently than would be expected by chance across a set of locations, R/cooccur v1.3 was used (Veech 2013; Griffith et al. 2016). To test if ASV abundance across the three dominant species was predicted by geospatial and environmental metrics, we ran linear models using the function lm in R/stats v4.2.3. To assess differences in community composition across tree species and insularity, a Bray–Curtis distance matrix was computed based on species‐level abundance data using the vegdist function from the package R/vegan (method ‘bray’). Permutational multivariate analysis of variance (PERMANOVA) was then conducted with the adonis2 function (R/vegan), using either tree species or insularity as the explanatory variable (999 permutations). Growth‐at‐temperature assay data were analysed by running linear models against longitude and latitude for each species detected at each temperature.

## 3 Results

A total of 149 ASVs were detected, of which 69 were attributed to the genus *Saccharomyces*. Up to 26 different ASVs were detected per tree, with on average 166,620 reads associated with ASVs per tree. No ASVs were detected in 3 out of 81 trees (one tree each in Blå Jungfrun, Björnstorp and Vårgårda‐1).

### 3.1 Fermentative Yeast Communities Across the Sampling Area Differ in Composition and Diversity

**FIGURE 2:**
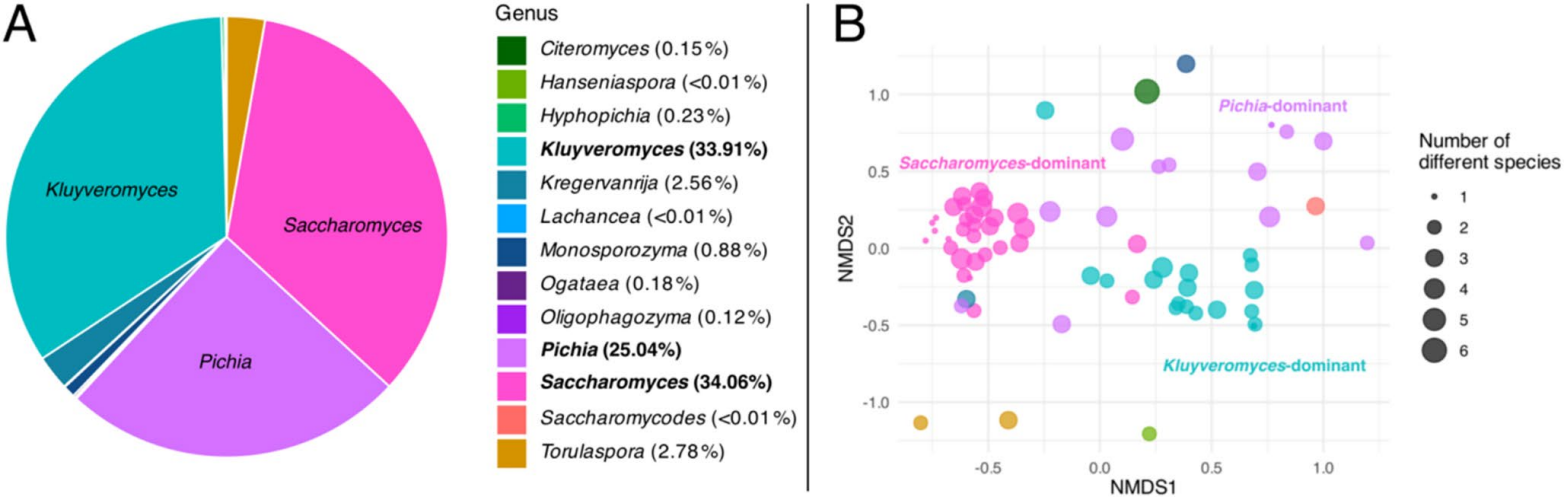
Overview of the 13 yeast genera detected on oak trees in Sweden. (A) Percentage of ASVs mapping to each genus across all samples. (B) Projection using non‐metric multidimensional scaling (NMDS), where each circle represents a single tree. Ordination is based on ASVs richness with colours indicating the dominant genus. Outlier samples LANY‐1, TAN‐1 and VASV‐1 were removed for display purposes.

First, we set out to test if fermentative yeast communities across the sampling area differ in their composition and diversity. We identified a total of 13 genera of yeasts (Figure 2A) belonging to four families across all samples (Debaryomycetaceae, Pichiaceae, Saccharomycetaceae and Saccharomycodaceae). Computing the sum of reads associated with ASVs detected across all samples for each family revealed that species belonging to Saccharomycetaceae were the most frequently detected (72.14%), followed by Pichiaceae (27.60%) (Figure S1). Among genera, *Saccharomyces* was the most common (34.06%), followed closely by two other genera of the Saccharomycetaceae family: *Kluyveromyces* (33.91%) and *Pichia* (25.04%) (Figure 2A). These three genera represented up to 93.01% of the total reads associated with ASVs, while each of the 11 remaining genera represented less than 3% of the reads associated with ASVs.

Samples were plotted using NMDS and coloured according to the most frequent genus (based on the read counts associated with ASVs) within each sample (Figure 2B). Three main clusters were revealed, which grouped together the samples that were dominated by species of either *Saccharomyces*, *Kluyveromyces* or *Pichia* (with a larger spread in the *Pichia* cluster). The proximity of points within each cluster suggests that fermentative yeast communities are more similar when sharing a common dominant genus. While there was no significant difference between the three clusters in species richness (median of 2.5 species), the number of yeast species per tree was slightly higher when the dominant genus was *Pichia* (Figure S2).

Up to 18 species were found across the 14 genera (Table 1), with between 1 and 69 ASVs associated with each species. Within the genus *Saccharomyces*, only *S. paradoxus* was detected. In the genus *Pichia*, we identified *P. kudriavzevii*, *P. mandshurica*, *P. membranifaciens* and *Pichia* sp. In the genus *Kluyveromyces*, we found *K. lactis* and *K. dobzhanskii*.

**TABLE 1:** Species detection across all genera.

Family	Genus	Species name	Occ.
Debaryomycetaceae	*Hyphopichia*	*Hyphopichia burtonii*	1
Pichiaceae	*Kregervanrija*	*Kregervanrija fluxuum*	2
Pichiaceae	*Pichia*	*Pichia kudriavzevii*	7
		*Pichia mandshurica*	1
		*Pichia membranifaciens*	37
		*Pichia* sp.	4
Saccharomycetaceae	*Citeromyces*	*Citeromyces matritensis*	1
Saccharomycetaceae	*Kluyveromyces*	*Kluyveromyces dobzhanskii*	50
		*Kluyveromyces lactis*	6
Saccharomycetaceae	*Lachancea*	*Lachancea kluyveri*	1
Saccharomycetaceae	*Monosporozyma*	*Monosporozyma servazzii*	1
Saccharomycetaceae	*Ogataea*	*Ogataea dorogensis*	2
		*Ogataea nonfermentans*	1
Saccharomycetaceae	*Oligophagozyma*	*Oligophagozyma castellii*	1
Saccharomycetaceae	*Saccharomyces*	*Saccharomyces paradoxus*	55
Saccharomycetaceae	*Torulaspora*	*Torulaspora delbrueckii*	11
Saccharomycodaceae	*Hanseniaspora*	*Hanseniaspora osmophila*	3
Saccharomycodaceae	*Saccharomycodes*	*Saccharomycodes ludwigii*	3

*Note:* Occurrence (occ.) represents the number of trees (out of 81) where species were detected with at least one ASV. Different colors highlight the three species with highest occurrences.

**FIGURE 3:**
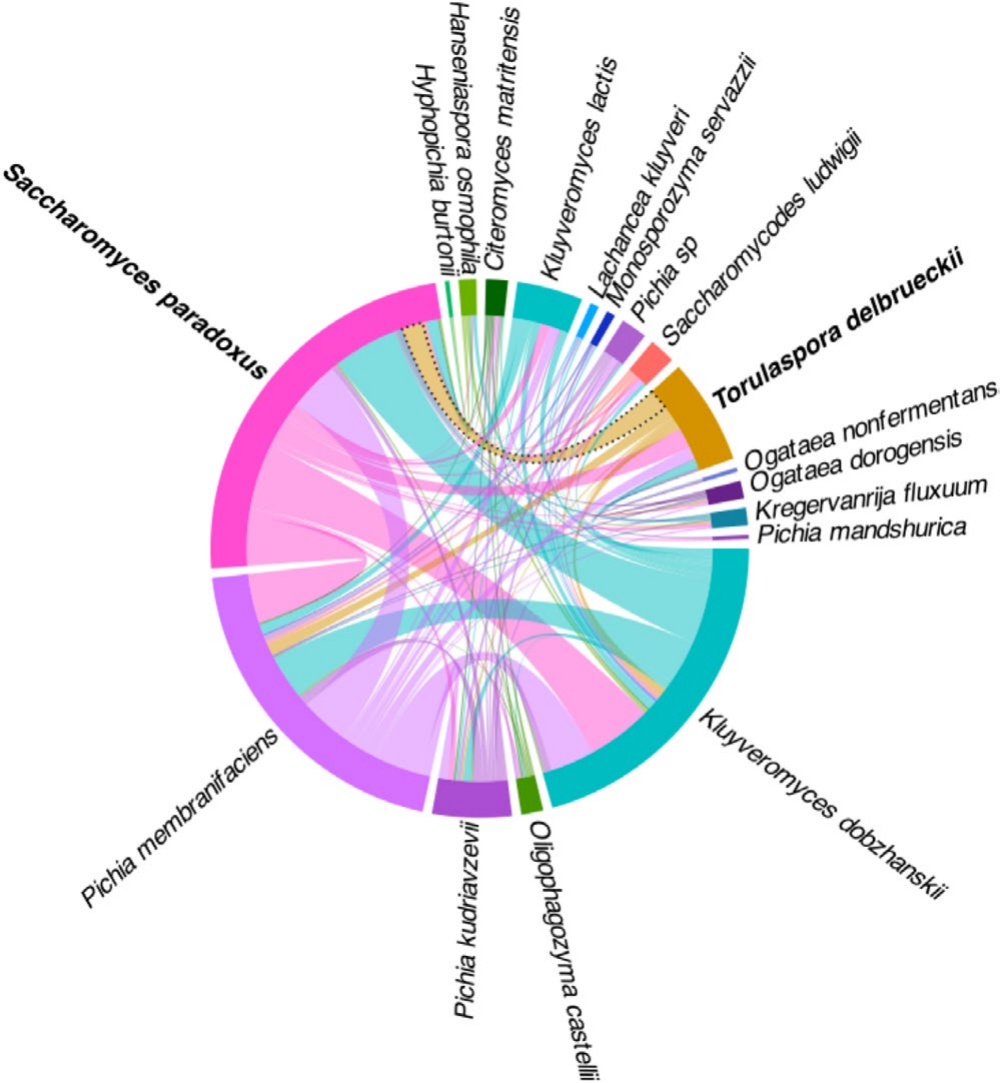
Chord diagram depicting yeast species co‐occurrence, based on shared presence across sampled trees. The connections (arcs) between species indicate how frequently they co‐occur, with thicker arcs representing more frequent co‐occurrences. Significant co‐occurrence was outlined with a dotted line, with species names involved in bold (binomial test, *p*‐value ‘greater than expected’ < 0.05).

Co‐occurrences of the 18 yeast species detected were visualised using a chord diagram (Figure 3). The thickness of the connecting lines between species reflects the frequency with which they co‐occur across the sampled locations (between zero and 34 times; Figure S3). The species that co‐occurred most often were *S. paradoxus*, *K. dobzhanskii* and *P. membranifaciens*. However, when comparing the observed frequency of co‐occurrence to what would be expected under a random null model (binomial test), only a single significant positive co‐occurrence was detected (dotted line, Figure 3; *p*‐value ‘greater than expected’ < 0.05, Figure S3) between *T.delbrueckii* and *S. paradoxus*, suggesting that these species are more likely to co‐occur, possibly sharing compatible ecological requirements or forming a symbiotic relationship. On the other hand, *Pichia* sp. and *S. paradoxus*, as well as *T. delbrueckii* and *K. dobzhanskii*, exhibited significant negative co‐occurrence (*p*‐value ‘smaller than expected’ < 0.05, Figure S3) suggesting that these species are less likely to be found in the same environment due distinct habitat preferences or niche competition.

### 3.2 Spatial Patterns: Yeast Species Dominance Is Correlated to Environmental Gradients

**FIGURE 4:**
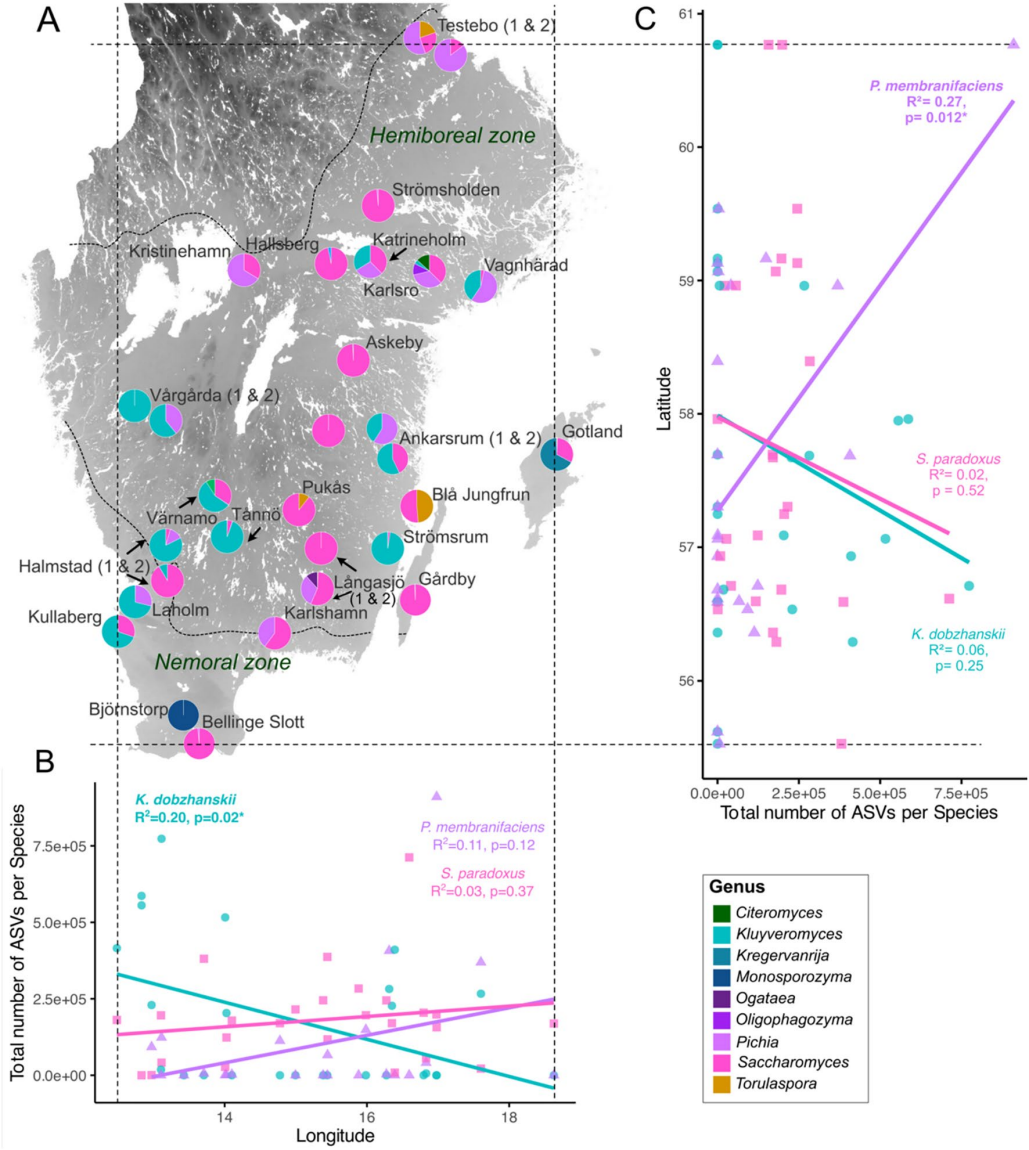
(A) Map of sampling locations with pie charts showing the proportion of ASVs (i.e., number of ASVs) found per yeast genus. For display purposes, only genera with > 2% of ASVs are shown in the legend. Dashed lines indicate the borders of hemiboreal and nemoral zones, in which oak trees were sampled. The upper line follows the northern latitudinal distribution limit of oak. (B) Longitude and (C) latitude correlated with the ASV abundance of the three most common species isolated across all locations (*Kluyveromyces dobzhanskii, Saccharomyces paradoxus* and *Pichia membranifaciens*). *R* ^2^ and *p*‐values are indicated for each linear model per genus (*p* < 0.05*, < 0.01**).

Next, we tested for spatial patterns in the abundance of the three most dominant yeast species detected, along latitudinal and longitudinal gradients. Dominant species were determined as having the highest read counts associated with ASVs across trees for a single location. We found that the abundance of ASVs significantly decreased with longitude (going from west to east) for *K. dobzhanskii* (linear model: *R* ^2^ = 0.21, *p* = 0.021), but not in *S. paradoxus* (*R* ^2^ = 0.03, *p* = 0.370) or *P. membranifaciens* (*R* ^2^ = 0.11, *p* = 0.119; Figure 4B). ASV abundance in *P*. *membranifaciens* significantly increased with latitude (going from south to north; linear model: *R* ^2^ = 0.27, *p* = 0.012), but not in *S. paradoxus* (*R* ^2^ = 0.02, *p* = 0.519) or *K. dobzhanskii* (*R* ^2^ = 0.06, *p* = 0.248; Figure 4C).

We then used a linear model to test whether variation in ASV abundance in *S. paradoxus* , *K. dobzhanskii* and *P. membranifaciens*, that is, ‘species’, was explained by ‘longitude’, ‘latitude’ and their interactions (minus the interactions Long:Lat and Species:Long:Lat), which was significant (*F*_8,66_ = 2.48, *p* = 0.020). Of the individual coefficients, ASV abundance was significantly affected by longitude overall (*t* = −2.42, *p* = 0.018) with significant differences between *P*. *membranifaciens* and *K. dobzhanskii* (*t* = −2.11, *p* = 0.039). Interactions between *S. paradoxus* versus *K*. *dobzhanskii* with longitude were also found to be significant (*t* = 2.54, *p* = 0.013).

Overall, these results suggest that *K*. *dobzhanskii* is significantly more abundant in the south‐west, that *P. membranifaciens* is most common in the north‐east and that *S. paradoxus* is most common in the centre of Sweden (Figure 4). Despite the frequent co‐occurrence of *S. paradoxus, K. dobzhanskii* and *P. membranifaciens* (Figure 3), this analysis highlights the respective dominance of different species over others along geospatial gradients.

**FIGURE 5:**
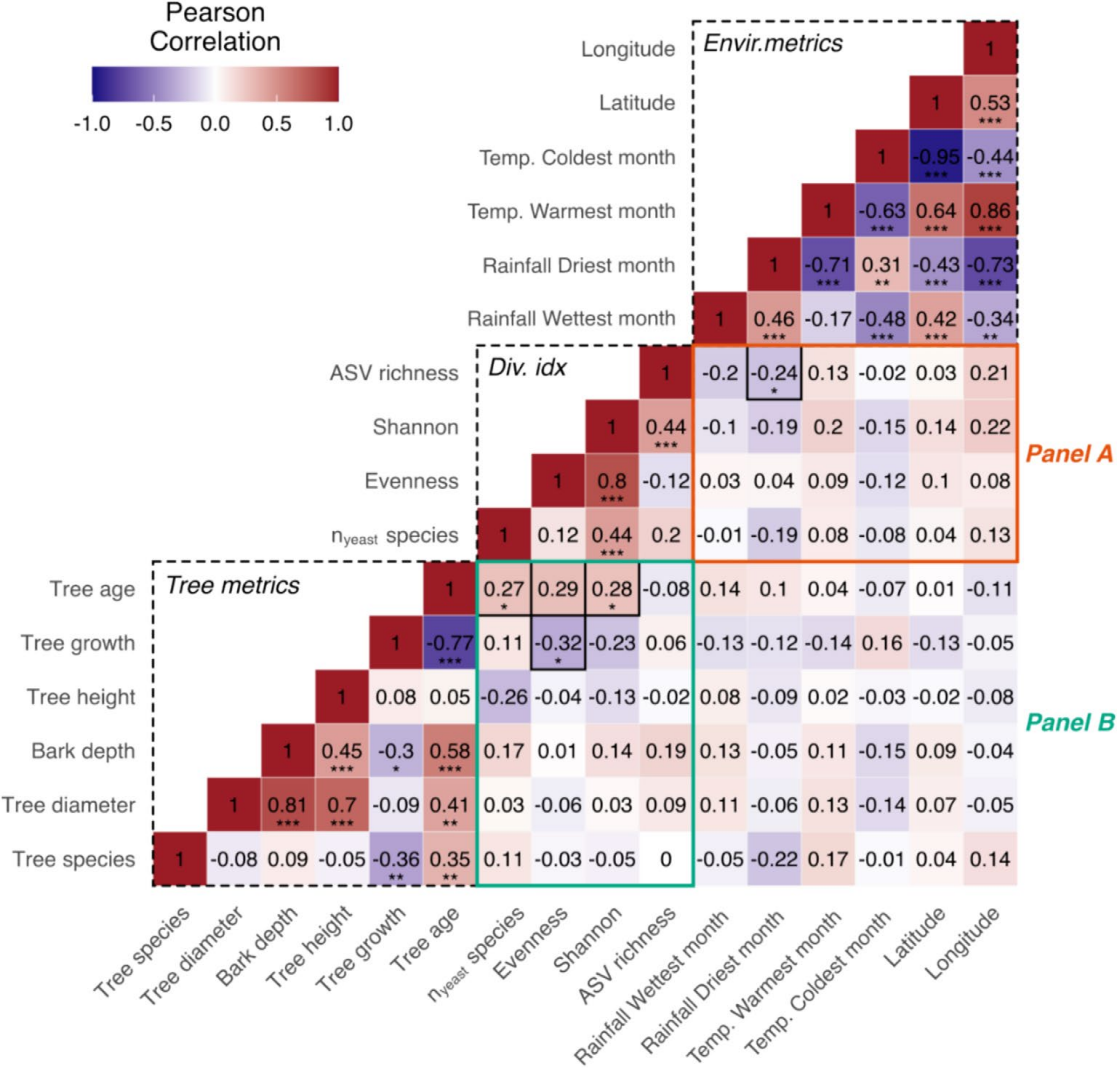
Correlation patterns between environmental variables, tree metrics and yeast diversity indices. Significance levels of Pearson correlations are indicated by asterisks (*p* < 0.05*, 0.01** and 0.001***).

**FIGURE 6:**
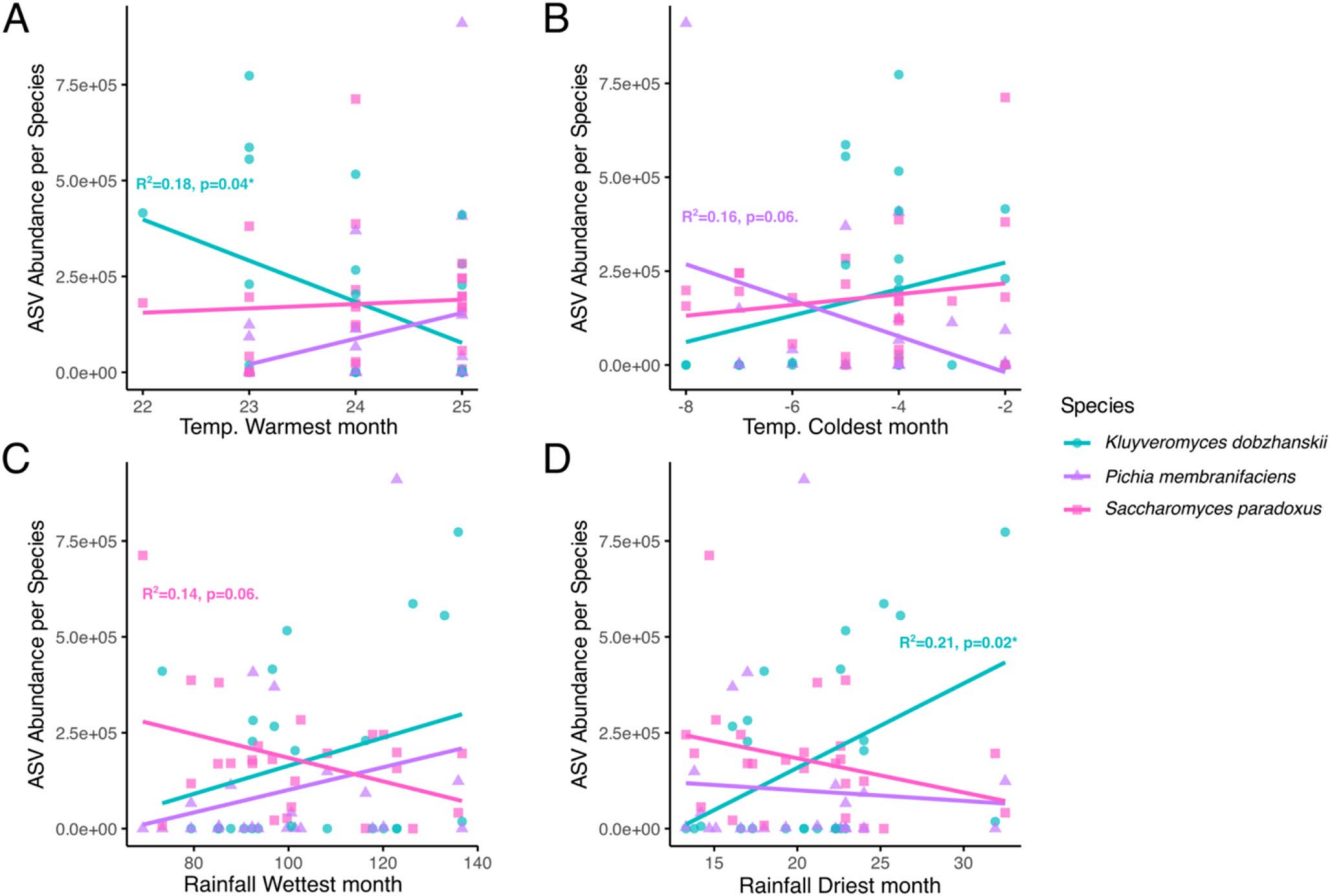
(A) Temperature during the warmest month, (B) temperature during the coldest month, (C) rainfall during the wettest month and (D) rainfall during the driest month correlated with the ASV abundance (total number of reads associated with ASVs) of the three most common species isolated across all locations (*Kluyveromyces dobzhanskii, Saccharomyces paradoxus* and *Pichia membranifaciens*). *R* ^2^ and *p*‐values are indicated for each linear model per genus if significant (or approaching significance).

As latitude increases going from south to north in our sampling area, temperature during the coldest month decreases (*r* = −0.95, df = 71, *p* < 2.2e−16) and rainfall during the wettest month increases (*r* = 0.64, df = 71, *p* = 9.05e−10; Figure 5). As longitude increases (going from west to east), temperature during the warmest month increases (*r* = 0.86, df = 71, *p* < 2.2e−16) and temperature during the coldest month decrease (*r* = −0.73, df = 71, *p* = 1.74e−13). We, therefore, tested whether these climate metrics (temperature during the warmest/coldest month and rainfall during the driest/wettest month) also predicted variation in yeast diversity (including ASV richness, Shannon, Evenness and the raw number of yeast species) and visualised the strength and direction of these correlations using a heatmap (Figure 5). We found that lower rainfall during the driest month negatively predicted ASV richness, suggesting that diversity is larger at drier sampling sites (*r* = −0.24, df = 71, *p* = 0.04, Figure 6A). Note here that this pattern may be largely caused by the high ASV richness observed for *Saccharomyces* (Figure S4).

Analysis of the impact of temperature and rainfall on ASV abundance at the ‘genus’ level showed that lower temperature during the warmest month significantly predicted an increase in *K. dobzhanskii's* ASV abundance (*R* ^2^ = 0.18, *p* = 0.035; Figure 6A), while lower temperature during the coldest month predicted higher ASV abundance in *P. membranifaciens* nearly significant (linear model: *R* ^2^ = 0.16 *p* = 0.062; Figure 6B). *S. paradoxus'* ASV abundance did not depend on either temperature metric. Lower rainfall during the wettest month nearly predicted ASV abundance in *S. paradoxus* (*R* ^2^ = 0.14, *p* = 0.063, Figure 6C), while higher rainfall during the driest month predicted higher *K*. *dobzhanskii* ASV abundance (*R* ^2^ = 0.21, *p* = 0.02; Figure 6D). Together, these patterns suggest that the colder temperatures and higher precipitation levels in the north‐eastern range limit of oak trees are well‐suited for *P. membranifaciens*, while the lower temperature during the warmest month and higher precipitation levels on the Swedish west coast are better suited for *K. dobzhanskii*. The absence of temperature effects on the distribution of *S. paradoxus* suggests that this species is more thermo‐generalist than the two other species.

### 3.3 Ecological Drivers of Yeast Diversity: Older Trees Have Richer and More Balanced Fermentative Yeast Communities

Third, we wanted to know if any ecological aspects of the oak tree hosts predicted yeast diversity. We tested for correlations between yeast diversity measures (including ASV richness, Shannon, Evenness and the raw number of yeast species per bark sample) and several oak tree variables (tree growth, age, height, diameter, bark depth and tree species (either *Q. robur* or *Q. petraea* )). Tree metrics are gathered in the bottom left corner of the heatmap (Figure 5). As expected, tree diameter was positively correlated with bark depth (*r* = 0.81, df = 57, *p* = 6.98e−15) and tree age (*r* = 0.41, df = 56, *p* = 0.001). A strong negative correlation was also found between tree age and tree growth (*r* = −0.77, df = 56, *p* = 9.17e−12), which indicates that older trees grow more slowly.

Species diversity (Shannon index, *r* = 0.28, df = 52, *p* = 0.042) and species richness (number of yeast species: *r* = 0.27, df = 52, *p* = 0.045) increased with tree age (Figure 5B). We also found that older, more slowly growing trees have a more balanced community composition, that is, species in the enriched fermentative yeast communities on older trees have more evenly distributed ASV abundances (correlation between tree growth and evenness: *r* = −0.32, df = 45, *p* = 0.03).

**FIGURE 7:**
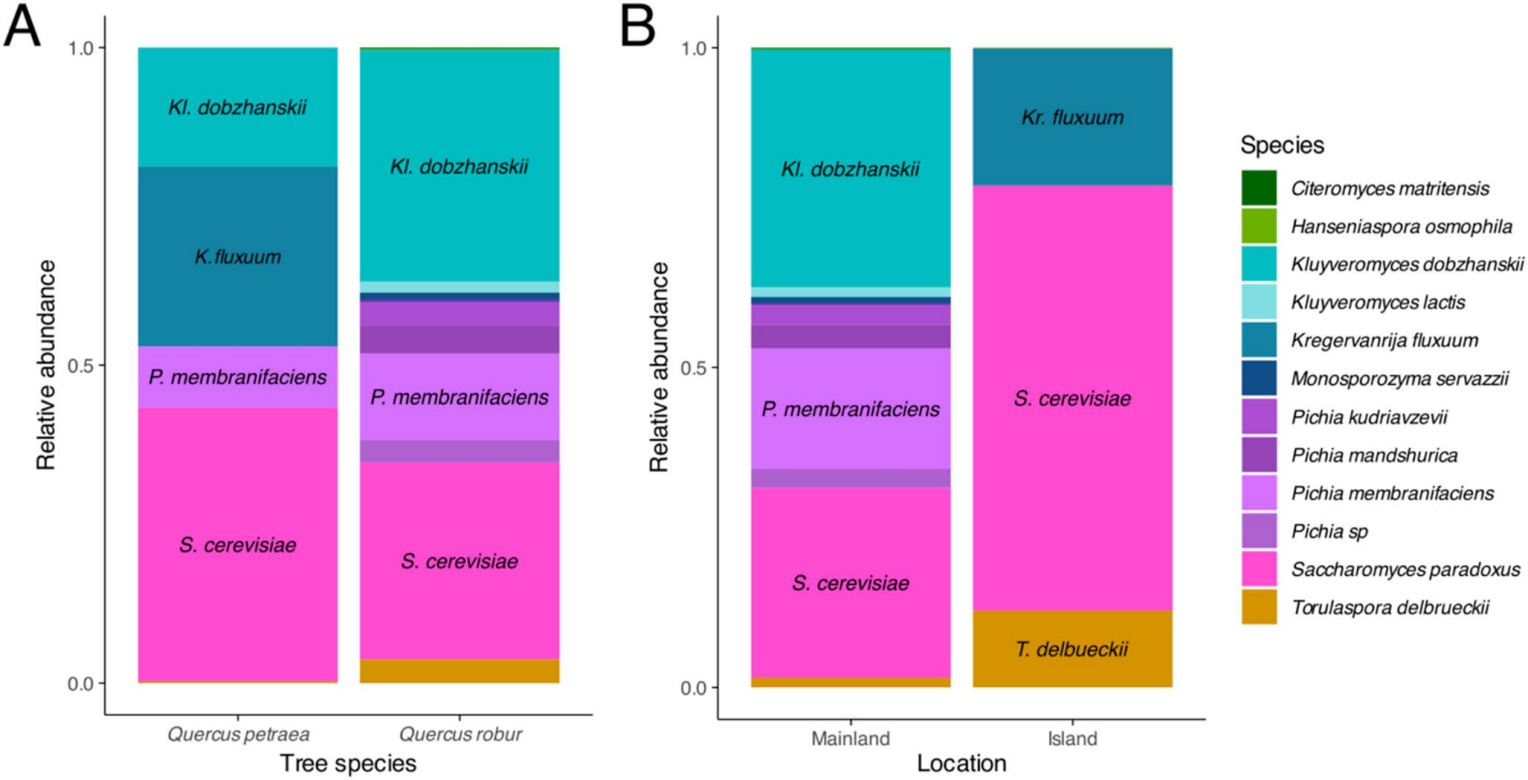
Relative abundance of yeast species depending on (A) host tree species and (B) insularity.

Although only 9 trees out of the 81 sampled were identified as *Q. petraea* , they show an interesting difference in yeast diversity, although not significantly (*F*_1,63_ = 0.412, *p* = 0.968), driven by one particular tree on the island of Gotland. Of the three trees sampled on Gotland, two were *Q. robur* and one was *Q. petraea* . The latter shows a very high abundance of *Kregervanrija fluxuum*, which, to our best knowledge, is the only reported occurrence so far of this species in Sweden (Figure 7A). The yeast diversity on islands off Sweden's east coast (Gotland, Gårdby and Blå Jungfrun) was different from the diversity found on the mainland, although not significantly (*F*_1,76_ = 0.851, *p* = 0.459). We observed an enrichment on these islands for *K.fluxuum*, but also *T. delbrueckii*, which was found to be highly abundant on a single tree sampled on the island of Blå Jungfrun (Figure 7B).

### 3.4 The Impact of Temperature on the Growth Performance of Wild Yeast Isolates

**FIGURE 8:**
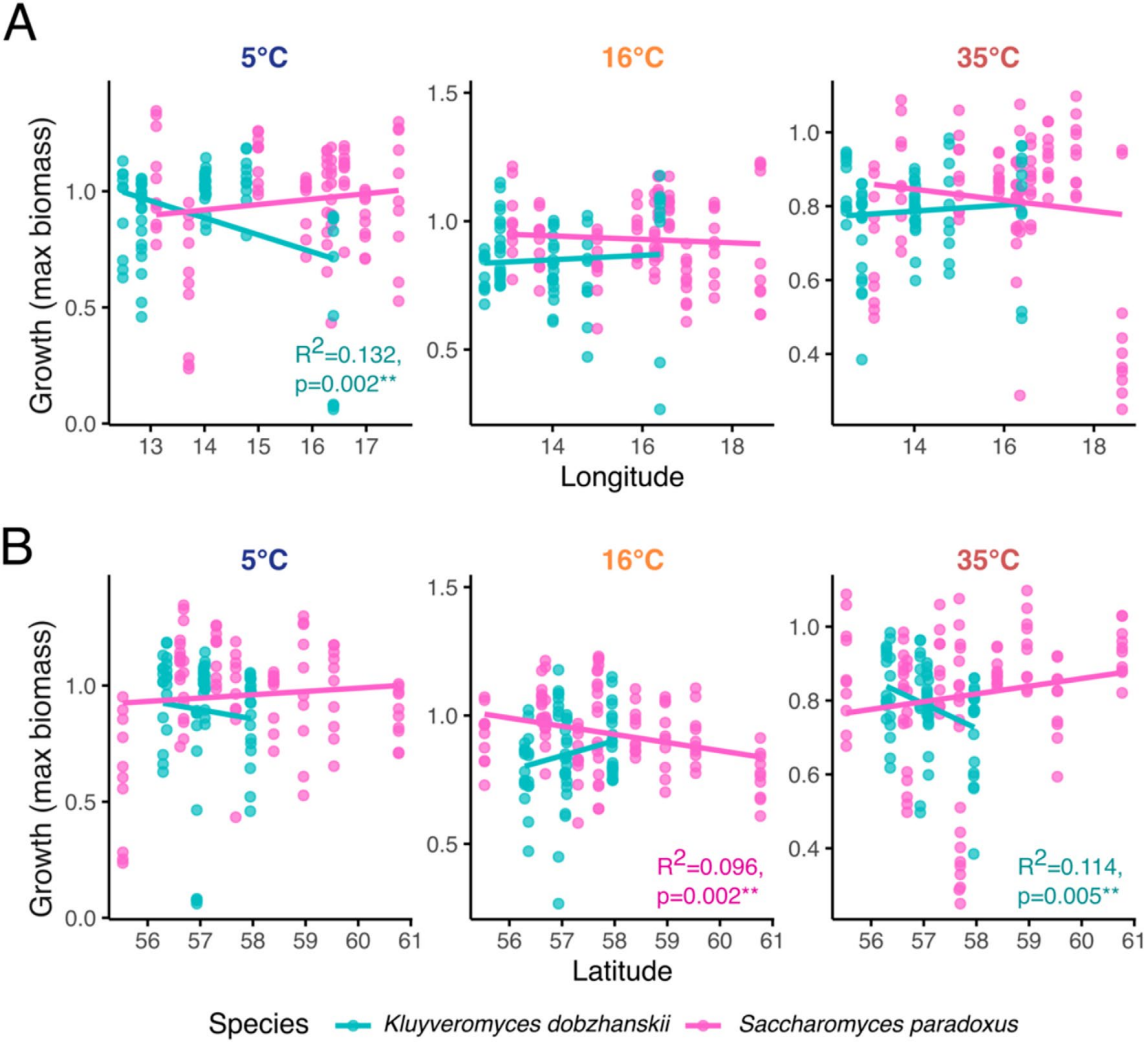
Growth (maximum biomass) of yeast strains at 5°C, 16°C and 35°C. Strains were identified by Sanger sequencing to belong to *Kluyveromyces dobzhanskii* and *Saccharomyces paradoxus* and plotted according to their sampling location's (A) longitude and (B) latitude. Each data point represents one of 10 technical replicate optical density measurements per strain and location. *R* ^2^ and *p*‐values are indicated for each linear model per genus if significant (*p* < 0.01**).

Finally, we tested whether the growth (maximum biomass) of the yeast strains we isolated across the sampling area was affected by temperature, using laboratory growth assays at three temperatures: 5°C, 16°C and 35°C. We found that culturing temperature significantly affected the growth of *K. dobzhanskii* collected from different longitudes and latitudes. *K. dobzhanskii* strains from western Sweden grew better than strains from the east at 5°C (*R* ^2^ = 0.132, *p* = 0.002; Figure 8A), while strains collected in southern Sweden grew better than strains from the north at 35°C (*R* ^2^ = 0.114, *p* = 0.005; Figure 8B). For *S. paradoxus*, southern strains grew better than strains from the north at 16°C (*R* ^2^ = 0.96, *p* = 0.002; Figure 8B) but no significant effects of latitude and longitude were found when growing *S. paradoxus* at extreme cold or hot temperatures.

## 4 Discussion

Temperate forests are a widespread global biome and a diversity hotspot for yeasts (Sniegowski et al. 2002; Sampaio and Gonçalves 2008; Robinson et al. 2016; Mozzachiodi et al. 2022; David et al. 2024). Fermentative yeasts play crucial roles in these ecosystems. They break down organic matter and contribute to nutrient recycling by converting sugars into ethanol, carbon dioxide and other metabolites such as esters and glycerol (Dashko et al. 2014). Despite their importance for nature and society, our knowledge of the distribution and diversity of fermentative yeasts across geographically and ecologically diverse natural environments is still scarce. Previous work suggests that oak trees are a frequent habitat for yeasts (Bowles and Lachance 1983; Sniegowski et al. 2002; Sampaio and Gonçalves 2008; Robinson et al. 2016; Mozzachiodi et al. 2022) but the diversity and abundance of yeasts in the northern range limit of their oak hosts have not been investigated. The temperate forests of Scandinavia are marked by a climate with strong seasonal variation between long, cold winters and mild summers. So far, most work in Scandinavia has focused on fungal communities on spruce (*Picea*) (Müller and Hallaksela 2000) and beech (*Fagus*) (Kubart et al. 2016; Asplund et al. 2019), but no previous study has investigated whether oak trees in this region carry similar yeast communities as trees further south in the *Quercus* range. Using DNA metabarcoding, we describe the species diversity and community composition of fermentative yeasts isolated from oak trees across a large area (covering 221,000 km^2^) in the temperate climate zone of Sweden and test whether climatic and ecological variables predict their distribution.

### 4.1 The Diversity and Community Composition of Oak‐Associated Yeasts in Sweden

We found three main clusters of oak‐associated yeast communities across southern Sweden, each dominated by species of either *Saccharomyces*, *Kluyveromyces* or *Pichia* (Figure 2B). Fermentative yeast communities across trees were more similar when sharing a common dominant genus. Generally, the dominance of one genus did not exclude a species from another genus to co‐occur in the same location, that is, there was no strong pattern of competitive exclusion. However, in some locations, only a single genus was detected (small dots in Figure 2B).

One species pair co‐occurred more frequently than expected by chance: *T. delbrueckii* and *S. paradoxus* (Figure 3), which suggests they have compatible ecological requirements or enter beneficial metabolic interactions. A study from temperate woodlands in North America has found *S. paradoxus* to often co‐occur with *S. cerevisiae* (Sweeney et al. 2004). Although *S. cerevisiae* is a cosmopolitan species due to its long history of domestication for alcoholic beverage production, we did not detect *S. cerevisiae* in Sweden. However, just as *S. cerevisiae, T. delbrueckii* has been domesticated for winemaking (Albertin et al. 2014) and has high tolerance to extremely low temperatures and freezing (Alves‐Araújo et al. 2004). Thus, *T. delbrueckii* may occupy a similar ecological niche in Sweden as *S. cerevisiae* elsewhere in the world, but may have adaptive advantages at higher latitudes due to its extreme cold tolerance. Other species pairs in our collection rarely or never overlapped in the same location (e.g., *T. delbrueckii* with *K. dobzhanskii* or *S. paradoxus* with *Pichia* sp.), perhaps due to competition or distinct habitat preferences. The positive co‐occurrence of *T. delbrueckii* with *S. paradoxus* and its negative co‐occurrence with *K. dobzhanskii*, reinforce our hypothesis that *Saccharomyces* and *Kluyveromyces* have different niches in Sweden (Figure 4).

We also identified yeasts used in industrial applications such as *Saccharomycodes*, which is used in the production of alcohol‐free beer (Montanari et al. 2009), *Kazachstania*, a yeast with potential use in biorefineries (Balarezo‐Cisneros et al. 2023), *Isaatchenkia*, a halophilic yeast with the capacity to grow at low‐pH conditions (Matsushika et al. 2016) and *Kregervanrija*, a yeast that weakly ferments sugars (Kurtzman 2011). Some of the species we detected are also found in human‐associated environments, for exampple, during the spontaneous fermentation of grape juice: non‐*Saccharomyces* yeasts such as *Hanseniospora*, *Pichia* and *Torulaspora* are predominant during the initial phase of alcoholic fermentation, before the conversion of sugars into ethanol by *Saccharomyce*s (Jolly et al. 2014). For instance, *P. membranifaciens* is commonly used in industrial applications because it produces ‘killer toxins’ that help control the growth of spoilage yeasts and filamentous fungi (Santos and Marquina 2004).

It is likely that other yeast species beyond the ones we detected here are also present on Swedish oak. The enrichment medium we used for strain isolation prior to DNA metabarcoding favours the growth of Saccharomycodaceae yeasts—and fermentative yeasts in general. Furthermore, ITS sequencing protocols can add additional bias, as they were shown to underestimate the presence of yeasts from the genus *Hanseniaspora* compared to 18S or 26S sequencing (De Filippis et al. 2017). Our collection of oak‐associated fermentative yeasts from southern Sweden, including 1150 cryo‐preserved strains across all sampling locations, is available upon request for future experimental work in ecology and evolution, or for potential uses in industry.

### 4.2 The Abundance and Distribution of Oak‐Associated Yeasts in Sweden

Our results reveal interesting patterns in the abundance and distribution of oak‐associated yeasts across a longitudinal gradient in southern Sweden, and suggest that climate variation from west to east and south to north, especially in temperature and precipitation (Figure 6), contributes to this. Both coasts experience an oceanic climate, with more extreme seasonal temperature fluctuations in the east than on the milder west coast (Köppen and Geiger 1930). The west coast, in particular, is characterised by a more humid climate. Going northwards, the climate gets colder and wetter and the vegetation changes from agricultural lands to deciduous forests, mixed forests and even some taiga. Our findings suggest genus‐specific responses to these climatic gradients (Figure 4B). *K. dobzhanskii* showed a strong negative correlation with longitude with higher abundance in western regions, while *P. membranifaciens* dominated in the east and *S. paradoxus* was more common in central areas. Compared to *P. membranifaciens, K*. *dobzhanskii* may be less tolerant to the larger temperature swings on the east coast. These differences in the spatial distribution of yeast genera across Sweden, together with their overlap in some locations (Figure 2B), suggest that niche‐defining ecological and climate factors are more likely to shape fermentative yeast community composition than competitive exclusion. In addition to climate, factors such as competition with other microbes, which we have not assessed here, also likely influence the observed distribution patterns (Kowallik et al. 2015). Although we sampled yeasts from bark, soil composition likely influences bark microbial communities as well, either indirectly via tree nutrient uptake (which is affected by soil pH and the availability of nitrogen and carbon), or directly through the dispersal of soil microbes to the bark surface (Brockett et al. 2012; Faticov et al. 2023; Mundra et al. 2021).

### 4.3 Effects of Temperature on the Growth of Yeast Isolates From the Wild

Previous studies have shown that species of both *Kluyveromyces* and *Pichia* have a wide range of critical thermal minima and maxima, from 5°C to 45°C (Dickson et al. 1979; Slininger et al. 1990; Nambu‐Nishida et al. 2017), suggesting that they are resilient to a large range of temperatures in the wild. Our assays revealed significant effects of temperature on the growth of fermentative yeasts collected from different longitudes and latitudes in Sweden, especially for *K. dobzhanskii* (Figure 8). The better cold performance (at 5°C) of *K. dobzhanskii* strains collected in the west compared to strains from the east suggests they are able to perform well in the relatively milder winter climate on the west coast, while the better performance of southern versus northern strains at hot conditions (35°C) indicates that southern strains are well adapted to the warm summer temperatures typical for this region, including occasional heat waves. This pattern is consistent with our finding that the more moderate climate in the southwest predicts a significantly higher abundance of *K*. *dobzhanskii* (Figure 4). In agreement with this, a study in North America showed that *K. dobzhanskii* was most frequently isolated at moderate temperatures (10°C–20°C) but less often at 30°C (Sylvester et al. 2015).

In *S. paradoxus*, the absence of significant effects of longitude and latitude on growth at extreme temperatures (Figure 8) is in line with the previously reported thermogeneralist performance of *S. paradoxus* (Sniegowski et al. 2002; Robinson et al. 2016)—the most abundant yeast species in our sampling area (Table 1 and Figure 2). *S. paradoxus* is, after *S. cerevisiae*, the most cosmopolitan species of *Saccharomyces* and is frequently isolated from trees, soil and bark of deciduous trees in the Northern Hemisphere (Charron et al. 2014). The ability of *Saccharomyces* to grow both at warm and cold temperatures, for instance through the production and subsequent use of glycerol as a cryo‐preservative (Hohmann 2002; Koh 2013), may give them a selective advantage over other fermentative yeasts in the generally cold climate of Sweden (Sweeney et al. 2004; Salvadó et al. 2011).

## 5 Conclusions

Our study provides insights into the diversity and distribution of fermentative yeasts in the northern range limit of their oak tree hosts. To our knowledge, our data provide the first evidence that (i) older oak trees supported greater yeast species richness and (ii) that the relative strain frequencies in each tree‐specific fermentative yeast community are more balanced, the older the tree is. This suggests that tree age plays an important role in establishing more stable microbial communities, allowing multiple species to coexist in similar abundance. We found that communities were largely dominated by species of the Saccharomycetaceae family, with notable longitudinal and latitudinal gradients in their composition, which are associated with climate variables, especially temperature and precipitation. Future research on the underlying genetic basis of thermal tolerance of these wild yeast isolates may shed light on their vulnerability to future climate change. This is important as changes in microbial community compositions can have cascading effects through the food web and potentially affect the diversity of entire ecosystems (Pörtner and Farrell 2008) and their services (Cavicchioli et al. 2019). Together, our findings contribute to a better understanding of the diversity of natural yeast communities and the drivers of their distribution in the environment.

## Author Contributions

**Javier Pinto:** conceptualization, methodology, formal analysis, investigation, visualization, data curation, writing – original draft, writing – review and editing. **Chloé Haberkorn:** conceptualization, investigation, visualization, methodology, formal analysis, data curation, writing – original draft, writing – review and editing. **Markus Franzén:** writing – review and editing. **Ayco J. M. Tack:** conceptualization, writing – review and editing. **Rike Stelkens:** conceptualization, writing – review and editing, writing – original draft, funding acquisition, project administration, resources, supervision, validation.

## Conflicts of Interest

The authors declare no conflicts of interest.

## Supporting information


**Data S1.** Supporting Information.

## Data Availability

The data that support the findings of this study are openly available in NCBI's Sequence Read Archive (SRA) database under BioProject PRJNA1114957. All the scripts used for data processing, analyses and figures are also openly available on GitHub: https://github.com/chaberko‐lbbe/yeast‐oaktree.

## References

[emi470110-bib-0001] Abarenkov, K. , R. H. Nilsson , K.‐H. Larsson , et al. 2024. “The UNITE Database for Molecular Identification and Taxonomic Communication of Fungi and Other Eukaryotes: Sequences, Taxa and Classifications Reconsidered.” Nucleic Acids Research 52, no. D1: D791–D797. 10.1093/nar/gkad1039. 37953409 PMC10767974

[emi470110-bib-0002] Albertin, W. , L. Chasseriaud , G. Comte , et al. 2014. “Winemaking and Bioprocesses Strongly Shaped the Genetic Diversity of the Ubiquitous Yeast Torulaspora Delbrueckii.” PLoS One 9, no. 4: e94246. 10.1371/journal.pone.0094246. 24718638 PMC3981792

[emi470110-bib-0003] Alsammar, H. F. , S. Naseeb , L. B. Brancia , R. T. Gilman , P. Wang , and D. Delneri . 2019. “Targeted Metagenomics Approach to Capture the Biodiversity of *Saccharomyces* Genus in Wild Environments.” Environmental Microbiology Reports 11, no. 2: 206–214. 10.1111/1758-2229.12724. 30507071 PMC6767435

[emi470110-bib-0004] Alves‐Araújo, C. , M. J. Almeida , M. J. Sousa , and C. Leão . 2004. “Freeze Tolerance of the Yeast Torulaspora Delbrueckii: Cellular and Biochemical Basis.” FEMS Microbiology Letters 240, no. 1: 7–14. 10.1016/j.femsle.2004.09.008. 15500973

[emi470110-bib-0005] Asplund, J. , H. Kauserud , M. Ohlson , and L. Nybakken . 2019. “Spruce and Beech as Local Determinants of Forest Fungal Community Structure in Litter, Humus and Mineral Soil.” FEMS Microbiology Ecology 95, no. 2: fiy232. 10.1093/femsec/fiy232. 30481314

[emi470110-bib-0006] Balarezo‐Cisneros, L. N. , S. Timouma , A. Hanak , A. Currin , F. Valle , and D. Delneri . 2023. “High Quality de Novo Genome Assembly of the Non‐Conventional Yeast Kazachstania Bulderi Describes a Potential Low pH Production Host for Biorefineries.” Communications Biology 6, no. 1: 918. 10.1038/s42003-023-05285-0. 37679437 PMC10484914

[emi470110-bib-0007] Battaglia, E. , I. Benoit , J. van den Brink , et al. 2011. “Carbohydrate‐Active Enzymes From the Zygomycete Fungus *Rhizopus oryzae*: A Highly Specialized Approach to Carbohydrate Degradation Depicted at Genome Level.” BMC Genomics 12: 1–12. 10.1186/1471-2164-12-38 PMC3032700 21241472

[emi470110-bib-0008] Blaalid, R. , S. Kumar , R. H. Nilsson , K. Abarenkov , P. M. Kirk , and H. Kauserud . 2013. “ITS1 Versus ITS2 as DNA Metabarcodes for Fungi.” Molecular Ecology Resources 13, no. 2: 218–224. 10.1111/1755-0998.12065. 23350562

[emi470110-bib-0009] Botha, A. 2011. “The Importance and Ecology of Yeasts in Soil.” Soil Biology and Biochemistry 43, no. 1: 1–8. 10.1016/j.soilbio.2010.10.001.

[emi470110-bib-0010] Bowles, J. M. , and M.‐A. Lachance . 1983. “Patterns of Variation in the Yeast Florae of Exudates in an Oak Community.” Canadian Journal of Botany 61, no. 12: 2984–2995.

[emi470110-bib-0011] Brockett, B. F. , C. E. Prescott , and S. J. Grayston . 2012. “Soil Moisture Is the Major Factor Influencing Microbial Community Structure and Enzyme Activities Across Seven Biogeoclimatic Zones in Western Canada.” Soil Biology & Biochemistry 44, no. 1: 9–20.

[emi470110-bib-0012] Cavicchioli, R. , W. J. Ripple , K. N. Timmis , et al. 2019. “Scientists' Warning to Humanity: Microorganisms and Climate Change.” Nature Reviews Microbiology 17, no. 9: 569–586. 31213707 10.1038/s41579-019-0222-5 PMC7136171

[emi470110-bib-0013] Charron, G. , J.‐B. Leducq , C. Bertin , A. K. Dubé , and C. R. Landry . 2014. “Exploring the Northern Limit of the Distribution of *Saccharomyces cerevisiae* and *Saccharomyces paradoxus* in North America.” FEMS Yeast Research 14, no. 2: 281–288. 24119009 10.1111/1567-1364.12100

[emi470110-bib-0014] Cubillos, F. A. , B. Gibson , N. Grijalva‐Vallejos , K. Krogerus , and J. Nikulin . 2019. “Bioprospecting for Brewers: Exploiting Natural Diversity for Naturally Diverse Beers.” Yeast 36, no. 6: 383–398. 30698853 10.1002/yea.3380

[emi470110-bib-0015] Dashko, S. , N. Zhou , C. Compagno , and J. Piškur . 2014. “Why, When, and How Did Yeast Evolve Alcoholic Fermentation?” FEMS Yeast Research 14, no. 6: 826–832. 24824836 10.1111/1567-1364.12161 PMC4262006

[emi470110-bib-0016] David, K. T. , M.‐C. Harrison , D. A. Opulente , et al. 2024. “Saccharomycotina Yeasts Defy Long‐Standing Macroecological Patterns.” Proceedings of the National Academy of Sciences of the United States of America 121, no. 10: e2316031121. 10.1073/pnas.2316031121. 38412132 PMC10927492

[emi470110-bib-0017] De Filippis, F. , M. Laiola , G. Blaiotta , and D. Ercolini . 2017. “Different Amplicon Targets for Sequencing‐Based Studies of Fungal Diversity.” Applied and Environmental Microbiology 83, no. 17: e00905. 28625991 10.1128/AEM.00905-17 PMC5561290

[emi470110-bib-0018] de Vries, R. P. , and J. Visser . 2001. “Aspergillus Enzymes Involved in Degradation of Plant Cell Wall Polysaccharides.” Microbiology and Molecular Biology Reviews 65, no. 4: 497–522. 10.1128/MMBR.65.4.497-522.2001. 11729262 PMC99039

[emi470110-bib-0019] Dickson, R. C. , L. R. Dickson , and J. S. Markin . 1979. “Purification and Properties of an Inducible Beta‐Galactosidase Isolated From the Yeast *Kluyveromyces lactis* .” Journal of Bacteriology 137, no. 1: 51–61. 33153 10.1128/jb.137.1.51-61.1979 PMC218417

[emi470110-bib-0020] Drobyshev, I. , S. Anderson , and K. Sonesson . 2007. “Crown Condition Dynamics of Oak in Southern Sweden 1988‐1999.” Environmental Monitoring and Assessment 134: 199–210. 17294275 10.1007/s10661-007-9610-9

[emi470110-bib-0021] Drobyshev, I. , M. Niklasson , O. Eggertsson , H. Linderson , and K. Sonesson . 2008. “Influence of Annual Weather on Growth of Pedunculate Oak in Southern Sweden.” Annals of Forest Science 65, no. 5: 1.

[emi470110-bib-0022] Faticov, M. , A. Abdelfattah , P. Hambäck , T. Roslin , and A. J. M. Tack . 2023. “Different Spatial Structure of Plant‐Associated Fungal Communities Above‐ and Belowground.” Ecology and Evolution 13, no. 5: e10065. 10.1002/ece3.10065. 37223309 PMC10200691

[emi470110-bib-0023] Ferreira, J. P. , I. Miranda , V. B. Sousa , and H. Pereira . 2018. “Chemical Composition of Barks From Quercus Faginea Trees and Characterization of Their Lipophilic and Polar Extracts.” PLoS One 13, no. 5: e0197135. 29763441 10.1371/journal.pone.0197135 PMC5953466

[emi470110-bib-0024] Fick, S. E. , and R. J. Hijmans . 2017. “WorldClim 2: New 1‐km Spatial Resolution Climate Surfaces for Global Land Areas.” International Journal of Climatology 37, no. 12: 4302–4315.

[emi470110-bib-0025] Griffith, D. M. , J. A. Veech , and C. J. Marsh . 2016. “Cooccur: Probabilistic Species Co‐Occurrence Analysis in R.” Journal of Statistical Software 69: 1–17.

[emi470110-bib-0026] Hohmann, S. 2002. “Osmotic Stress Signaling and Osmoadaptation in Yeasts.” Microbiology and Molecular Biology Reviews 66, no. 2: 300–372. 12040128 10.1128/MMBR.66.2.300-372.2002 PMC120784

[emi470110-bib-0027] Holmes, R. L. 1983. “Computer‐Assisted Quality Control in Tree‐Ring Dating and Measurement.” Tree‐Ring Bulletin 43: 69–78.

[emi470110-bib-0028] Johansson, V. , K.‐O. Bergman , H. Lättman , and P. Milberg . 2009. “Tree and Site Quality Preferences of Six Epiphytic Lichens Growing on Oaks in Southeastern Sweden.” Annales Botanici Fennici 46, no. 6: 496–506. 10.5735/085.046.0602.

[emi470110-bib-0029] Johnson, S. E. , and M. D. Abrams . 2009. “Age Class, Longevity and Growth Rate Relationships: Protracted Growth Increases in Old Trees in the Eastern United States.” Tree Physiology 29, no. 11: 1317–1328. 19734547 10.1093/treephys/tpp068

[emi470110-bib-0030] Jolly, N. P. , C. Varela , and I. S. Pretorius . 2014. “Not Your Ordinary Yeast: Non‐*Saccharomyces* Yeasts in Wine Production Uncovered.” FEMS Yeast Research 14, no. 2: 215–237. 24164726 10.1111/1567-1364.12111

[emi470110-bib-0031] Jonsson, B. G. , M. Ekström , P.‐A. Esseen , A. Grafström , G. Ståhl , and B. Westerlund . 2016. “Dead Wood Availability in Managed Swedish Forests–Policy Outcomes and Implications for Biodiversity.” Forest Ecology and Management 376: 174–182.

[emi470110-bib-0032] Koh, C. M. 2013. “Storage of Bacteria and Yeast.” Methods in Enzymology 533: 15–21. 24182914 10.1016/B978-0-12-420067-8.00002-7

[emi470110-bib-0033] Köppen, W. , and R. Geiger . 1930. Handbuch der klimatologie. Vol. 1. Gebrüder Borntraeger.

[emi470110-bib-0034] Kowallik, V. , E. Miller , and D. Greig . 2015. “The Interaction of *Saccharomyces paradoxus* With Its Natural Competitors on Oak Bark.” Molecular Ecology 24, no. 7: 1596–1610. 25706044 10.1111/mec.13120 PMC4405091

[emi470110-bib-0035] Kubart, A. , R. Vasaitis , J. Stenlid , and A. Dahlberg . 2016. “Fungal Communities in Norway Spruce Stumps Along a Latitudinal Gradient in Sweden.” Forest Ecology and Management 371: 50–58.

[emi470110-bib-0036] Kurtzman, C. P. 2011. “Kregervanrija Kurtzman (2006).” In The Yeasts, edited by C. Kurtzman , J. W. Fell , and T. Boekhout , 497–501. Elsevier.

[emi470110-bib-0038] Löf, M. , J. Brunet , A. Filyushkina , M. Lindbladh , J. P. Skovsgaard , and A. Felton . 2016. “Management of Oak Forests: Striking a Balance Between Timber Production, Biodiversity and Cultural Services.” International Journal of Biodiversity Science, Ecosystem Services & Management 12, no. 1–2: 59–73.

[emi470110-bib-0039] Matsushika, A. , K. Negi , T. Suzuki , T. Goshima , and T. Hoshino . 2016. “Identification and Characterization of a Novel Issatchenkia Orientalis GPI‐Anchored Protein, IoGas1, Required for Resistance to Low pH and Salt Stress.” PLoS One 11, no. 9: e0161888. 27589271 10.1371/journal.pone.0161888 PMC5010203

[emi470110-bib-0040] Montanari, L. , O. Marconi , H. Mayer , and P. Fantozzi . 2009. “Production of Alcohol‐Free Beer.” In Beer in Health and Disease Prevention, 61–75. Elsevier.

[emi470110-bib-0041] Mozzachiodi, S. , F. Bai , P. Baldrian , et al. 2022. “Yeasts From Temperate Forests.” Yeast 39, no. 1–2: 4–24. 35146791 10.1002/yea.3699

[emi470110-bib-0042] Müller, M. M. , and A.‐M. Hallaksela . 2000. “Fungal Diversity in Norway Spruce: A Case Study.” Mycological Research 104, no. 9: 1139–1145.

[emi470110-bib-0043] Mundra, S. , O. J. Kjønaas , L. N. Morgado , A. K. Krabberød , Y. Ransedokken , and H. Kauserud . 2021. “Soil Depth Matters: Shift in Composition and Inter‐Kingdom Co‐Occurrence Patterns of Microorganisms in Forest Soils.” FEMS Microbiology Ecology 97, no. 3: fiab022. 33547899 10.1093/femsec/fiab022 PMC7948073

[emi470110-bib-0044] Nambu‐Nishida, Y. , K. Nishida , T. Hasunuma , and A. Kondo . 2017. “Development of a Comprehensive Set of Tools for Genome Engineering in a Cold‐and Thermo‐Tolerant *Kluyveromyces marxianus* Yeast Strain.” Scientific Reports 7, no. 1: 8993. 28827530 10.1038/s41598-017-08356-5 PMC5566861

[emi470110-bib-0045] Nilsson, R. H. , K.‐H. Larsson , A. F. S. Taylor , et al. 2019. “The UNITE Database for Molecular Identification of Fungi: Handling Dark Taxa and Parallel Taxonomic Classifications.” Nucleic Acids Research 47, no. D1: D259–D264. 10.1093/nar/gky1022. 30371820 PMC6324048

[emi470110-bib-0046] Oksanen, J. , F. G. Blanchet , R. Kindt , et al. 2020. “vegan: Community Ecology Package. R Package Version 2.5–7.” Nov 28, 2020. https://vegandevs.github.io/vegan/.

[emi470110-bib-0047] Opulente, D. A. , A. L. LaBella , M.‐C. Harrison , et al. 2024. “Genomic Factors Shape Carbon and Nitrogen Metabolic Niche Breadth Across Saccharomycotina Yeasts.” Science 384, no. 6694: eadj4503. 10.1126/science.adj4503. 38662846 PMC11298794

[emi470110-bib-0048] Pörtner, H. O. , and A. P. Farrell . 2008. “Physiology and Climate Change.” Science 322, no. 5902: 690–692. 18974339 10.1126/science.1163156

[emi470110-bib-0049] Rinn, F. 2003. TSAP‐Win. Time Series Analysis and Presentation for Dendrochronology and 409 Related Applications. Rinntech Inc.

[emi470110-bib-0050] Robinson, H. A. , A. Pinharanda , and D. Bensasson . 2016. “Summer Temperature Can Predict the Distribution of Wild Yeast Populations.” Ecology and Evolution 6, no. 4: 1236–1250. 26941949 10.1002/ece3.1919 PMC4761769

[emi470110-bib-0051] Romero‐Olivares, A. , E. W. Morrison , A. Pringle , and S. D. Frey . 2021. “Linking Genes to Traits in Fungi.” Microbial Ecology 82, no. 1: 145–155. 33483845 10.1007/s00248-021-01687-x PMC8282587

[emi470110-bib-0052] Salvadó, Z. , F. N. Arroyo‐López , J. M. Guillamón , G. Salazar , A. Querol , and E. Barrio . 2011. “Temperature Adaptation Markedly Determines Evolution Within the Genus *Saccharomyces* .” Applied and Environmental Microbiology 77, no. 7: 2292–2302. 10.1128/AEM.01861-10. 21317255 PMC3067424

[emi470110-bib-0053] Sampaio, J. P. , and P. Gonçalves . 2008. “Natural Populations of *Saccharomyces kudriavzevii* in Portugal Are Associated With Oak Bark and Are Sympatric With *S. cerevisiae* and *S. Paradoxus* .” Applied and Environmental Microbiology 74, no. 7: 2144–2152. 10.1128/AEM.02396-07. 18281431 PMC2292605

[emi470110-bib-0054] Santos, A. , and D. Marquina . 2004. “Killer Toxin of Pichia Membranifaciens and Its Possible Use as a Biocontrol Agent Against Grey Mould Disease of Grapevine.” Microbiology 150, no. 8: 2527–2534. 15289549 10.1099/mic.0.27071-0

[emi470110-bib-0055] Shannon, C. E. , and W. Weaver . 1963. The Mathematical Theory of Communication. University of Illinois Press.

[emi470110-bib-0056] Slininger, P. , R. Bothast , M. Ladisch , and M. Okos . 1990. “Optimum pH and Temperature Conditions for Xylose Fermentation by *Pichia stipitis* .” Biotechnology and Bioengineering 35, no. 7: 727–731. 18592569 10.1002/bit.260350710

[emi470110-bib-0057] SMHI . 2023. “Swedish Meteorological and Hydrological Institute Open Data, Swedish Temperature Record.” May, 2025. https://www.smhi.se/kunskapsbanken/meteorologi/svenska‐temperaturrekord/svenska‐temperaturrekord‐1.5792.

[emi470110-bib-0058] Sniegowski, P. D. , P. G. Dombrowski , and E. Fingerman . 2002. “ *Saccharomyces cerevisiae* and *Saccharomyces paradoxus* Coexist in a Natural Woodland Site in North America and Display Different Levels of Reproductive Isolation From European Conspecifics.” FEMS Yeast Research 1, no. 4: 299–306. 12702333 10.1111/j.1567-1364.2002.tb00048.x

[emi470110-bib-0059] Straub, D. , N. Blackwell , A. Langarica‐Fuentes , A. Peltzer , S. Nahnsen , and S. Kleindienst . 2020. “Interpretations of Environmental Microbial Community Studies Are Biased by the Selected 16S rRNA (Gene) Amplicon Sequencing Pipeline.” Frontiers in Microbiology 11: 550420. 33193131 10.3389/fmicb.2020.550420 PMC7645116

[emi470110-bib-0060] Sweeney, J. Y. , H. A. Kuehne , and P. D. Sniegowski . 2004. “Sympatric Natural *Saccharomyces cerevisiae* and *S. paradoxus* Populations Have Different Thermal Growth Profiles.” FEMS Yeast Research 4, no. 4–5: 521–525. 14734033 10.1016/S1567-1356(03)00171-5

[emi470110-bib-0061] Sylvester, K. , Q.‐M. Wang , B. James , R. Mendez , A. B. Hulfachor , and C. T. Hittinger . 2015. “Temperature and Host Preferences Drive the Diversification of *Saccharomyces* and Other Yeasts: A Survey and the Discovery of Eight New Yeast Species.” FEMS Yeast Research 15, no. 3: fov002. 25743785 10.1093/femsyr/fov002

[emi470110-bib-0062] Tedersoo, L. , M. Bahram , L. Zinger , et al. 2022. “Best Practices in Metabarcoding of Fungi: From Experimental Design to Results.” Molecular Ecology 31, no. 10: 2769–2795. 10.1111/mec.16460. 35395127

[emi470110-bib-0063] Treseder, K. K. , and J. T. Lennon . 2015. “Fungal Traits That Drive Ecosystem Dynamics on Land.” Microbiology and Molecular Biology Reviews 79, no. 2: 243–262. 25971588 10.1128/MMBR.00001-15 PMC4429240

[emi470110-bib-0064] Veech, J. A. 2013. “A Probabilistic Model for Analysing Species Co‐Occurrence.” Global Ecology and Biogeography 22, no. 2: 252–260.

[emi470110-bib-0065] Větrovský, T. , P. Kohout , M. Kopecký , et al. 2019. “A Meta‐Analysis of Global Fungal Distribution Reveals Climate‐Driven Patterns.” Nature Communications 10, no. 1: 5142. 10.1038/s41467-019-13164-8 PMC6853883 31723140

[emi470110-bib-0066] Viljoen, B. C. 2006. “Yeast Ecological Interactions. Yeast'Yeast, Yeast'Bacteria, Yeast'Fungi Interactions and Yeasts as Biocontrol Agents.” In Yeasts in Food and Beverages, edited by A. Querol and G. Flee , 83–110. Springer Berlin Heidelberg.

[emi470110-bib-0067] Villarreal, P. , P. A. Quintrel , S. Olivares‐Muñoz , J. J. Ruiz , R. F. Nespolo , and F. A. Cubillos . 2022. “Identification of New Ethanol‐Tolerant Yeast Strains With Fermentation Potential From Central Patagonia.” Yeast 39, no. 1–2: 128–140. 34406697 10.1002/yea.3662

[emi470110-bib-0068] White, T. J. , T. Bruns , S. Lee , and J. Taylor . 1990. “38—Amplification and Direct Sequencing of Fungal Ribosomal RNA Genes for Phylogenetics.” In PCR Protocols, edited by M. A. Innis , D. H. Gelfand , J. J. Sninsky , and T. J. White , 315–322. Academic Press. 10.1016/B978-0-12-372180-8.50042-1.

[emi470110-bib-0069] Wilsey, B. J. , and C. Potvin . 2000. “Biodiversity and Ecosystem Functioning: Importance of Species Evenness in an Old Field.” Ecology 81, no. 4: 887–892.

